# Identification of gene signatures and molecular mechanisms underlying the mutual exclusion between psoriasis and leprosy

**DOI:** 10.1038/s41598-024-52783-0

**Published:** 2024-01-25

**Authors:** You-Wang Lu, Rong-Jing Dong, Lu-Hui Yang, Jiang Liu, Ting Yang, Yong-Hong Xiao, Yong-Jun Chen, Rui-Rui Wang, Yu-Ye Li

**Affiliations:** 1 Department of Dermatology and Venereology, First Affiliated Hospital of Kunming Medical University, Kunming, 650032 China; 2 Hubei Provincial Key Laboratory of Occurrence and Intervention of Kidney Diseases, Medical College, Hubei Polytechnic University, Huangshi, China; 3 College of Pharmaceutical Sciences, Yunnan University of Traditional Chinese Medicine, Kunming, 650500 China; 4 Department of Dermatology, Huangshi Central Hospital, Affiliated Hospital of Hubei Polytechnic University, Edong Healthcare Group, Huangshi, China; 5 Department of Reproduction and Genetics, First Affiliated Hospital of Kunming Medical University, Kunming, 650032 China

**Keywords:** Data mining, Skin diseases, Biomarkers, Pathogenesis

## Abstract

Leprosy and psoriasis rarely coexist, the specific molecular mechanisms underlying their mutual exclusion have not been extensively investigated. This study aimed to reveal the underlying mechanism responsible for the mutual exclusion between psoriasis and leprosy. We obtained leprosy and psoriasis data from ArrayExpress and GEO database. Differential expression analysis was conducted separately on the leprosy and psoriasis using DEseq2. Differentially expressed genes (DEGs) with opposite expression patterns in psoriasis and leprosy were identified, which could potentially involve in their mutual exclusion. Enrichment analysis was performed on these candidate mutually exclusive genes, and a protein–protein interaction (PPI) network was constructed to identify hub genes. The expression of these hub genes was further validated in an external dataset to obtain the critical mutually exclusive genes. Additionally, immune cell infiltration in psoriasis and leprosy was analyzed using single-sample gene set enrichment analysis (ssGSEA), and the correlation between critical mutually exclusive genes and immune cells was also examined. Finally, the expression pattern of critical mutually exclusive genes was evaluated in a single-cell transcriptome dataset. We identified 1098 DEGs in the leprosy dataset and 3839 DEGs in the psoriasis dataset. 48 candidate mutually exclusive genes were identified by taking the intersection. Enrichment analysis revealed that these genes were involved in cholesterol metabolism pathways. Through PPI network analysis, we identified APOE, CYP27A1, FADS1, and SOAT1 as hub genes. APOE, CYP27A1, and SOAT1 were subsequently validated as critical mutually exclusive genes on both internal and external datasets. Analysis of immune cell infiltration indicated higher abundance of 16 immune cell types in psoriasis and leprosy compared to normal controls. The abundance of 6 immune cell types in psoriasis and leprosy positively correlated with the expression levels of APOE and CYP27A1. Single-cell data analysis demonstrated that critical mutually exclusive genes were predominantly expressed in Schwann cells and fibroblasts. This study identified APOE, CYP27A1, and SOAT1 as critical mutually exclusive genes. Cholesterol metabolism pathway illustrated the possible mechanism of the inverse association of psoriasis and leprosy. The findings of this study provide a basis for identifying mechanisms and therapeutic targets for psoriasis.

## Introduction

With the development of economic levels, the incidence of many infectious diseases such as tuberculosis and leprosy is gradually decreasing, while the incidence of immune-inflammatory diseases such as psoriasis, atopic dermatitis, systemic lupus erythematosus, etc., is gradually increasing. Psoriasis is a common chronic inflammatory skin disease characterized by increased epidermal hyperplasia, erythematous papules, and plaques^1^. While not life-threatening, psoriasis significantly impacts patients’ quality of life due to its long duration and recurrent episodes^2^. The prevalence of psoriasis varies worldwide, ranges from 0.14 to 1.99%^3^. The exact etiology of psoriasis remains unclear, but it is believed to involve multiple factors such as infection, genetics, immune system dysfunction, and metabolic abnormalities^4,5^. While, leprosy, also known as Hansen's disease, is a chronic granulomatous disease caused by infection with *Mycobacterium leprae*, primarily affecting the peripheral nerves and skin^6^. These pathogens typically reside in the subepidermal zone, Schwann cells, macrophages, and perifollicular regions^7^, leading to skin and peripheral nerve damage^8^. Leprosy serves as an ideal model for studying immune pathways in disease pathogenesis.

The relationship between leprosy and psoriasis was enigmatic^9,10^. The term "lepra" in the Bible was historically used to describe a condition that we now recognize as psoriasis. The confusion between these two diseases persisted for nearly 19 centuries before it was realized that they are indeed distinct and separate conditions^11^. On the other hand, the coexistence of leprosy and psoriasis is rare, yet the skin lesions of the two conditions are remarkably similar^9^. The first report on this phenomena dates back four decades^12^. Leprosy patients are less susceptible to psoriasis. A survey of 45,661 leprosy patients reported a psoriasis prevalence rate of 0.014% in the cohort^13^. Currently, only sporadic case reports exist regarding patients with both psoriasis and leprosy^9,11,14–18^. In these two conditions, one disease may confer protection against the development of the other, or there might be shared mechanisms influencing the progression of both diseases. Gaining a comprehensive understanding how one disease prevents or ameliorates the other holds great significance for psoriasis treatment.

Previous studies have suggested that the mutual exclusion of psoriasis and leprosy may be linked to genetic factors, immune responses, and neurological damage^13^. Studies have revealed that leprosy and psoriasis share numerous susceptible genes, including HLA-Cw06, HLA-DRB104, IL23R, IL12B, and other genes^19–22^. Some of these susceptible genes play opposing roles in both diseases, for instance, the HLA-Cw06 allele can enhance the resistance of psoriasis patients to bacterial infections^21,23^, while HLA-Cw*06 allele are significantly negatively correlated with leprosy^19,24^. Moreover, a recent genome-wide association study (GWAS) found ZFP36L1 gene play opposing roles in the two diseases^25^, the protective ZFP36L1 genotypes against leprosy are the risk genotypes for psoriasis^25^. This indirectly suggests a genetic antagonism in susceptibility between psoriasis and leprosy. Therefore, some scholars propose a hypothesis that resistance to leprosy may confer an evolutionary advantage for the expansion of genotypes associated with psoriasis^10^.

Furthermore, psoriasis is characterized by significantly enhanced innate immunity and highly activated adaptive immune responses, including Th1 and Th17 responses, along with elevated levels of inflammatory factors such as TNF-α, IL-17, etc. These factors are essential for the body’s control of invasion and proliferation by *Mycobacterium leprae*^26,27^. Thus the hyperactive immune state observed in psoriasis enables patients to better defend against *Mycobacterium leprae*. infection^27^.

This may be the immunological reason for the "exclusion" between psoriasis and leprosy. In addition, current research suggests that neuroinflammation is an essential part of the mechanism underlying psoriasis^28^. While, the damage and dysfunction caused by *Mycobacterium leprae* to peripheral nerves, along with a decrease in neural secretion, appear to be another immunological reason why leprosy patients do not develop psoriasis.

Nowadays, there have been few studies investigate the mutual exclusion between leprosy and psoriasis based on bioinformatics analysis. The potential regulatory mechanisms between the two diseases remain elusive.

Comparative transcriptome analysis offers a potential avenue for gaining insights into the mechanisms underlying mutual exclusion between psoriasis and leprosy. Thus, the objective of this study was to identify hub genes and related signaling pathways that are mutually exclusive between psoriasis and leprosy. We analyzed two gene expression datasets, namely GSE54456 and MTAB-10318, and employed integrated bioinformatics and enrichment analysis to identify mutually exclusive genes and their functions in psoriasis and leprosy. Furthermore, we utilized the STRING database and Cytoscape software to construct protein–protein interaction (PPI) networks and identify hub genes. Finally, we identified three crucial hub genes and examined their correlation with immune cells, followed by evaluating their expression pattern at the single-cell level. This study is expected to provide novel insights into the inverse association of psoriasis and leprosy.

## Materials and methods

### Data sources

Information on the annual incidence rate of leprosy and psoriasis were obtained from GBD 2019 database with the Global Health Data Exchange (GHDx) query tool (http://ghdx.healthdata.org/gbd-results-tool).

The leprosy transcriptome dataset E-MTAB-10318 (33 leprosy samples and 9 normal control samples) was obtained from the ArrayExpress database (https://www.ebi.ac.uk/biostudies/arrayexpress/). Psoriasis transcriptome datasets were obtained from the GEO database (https://www.ncbi.nlm.nih.gov/geo/), including GSE54456 (92 psoriasis samples and 82 normal control tissue samples), validation set GSE13355 (58 psoriasis samples and 58 non-lesion samples), GSE30999 (85 psoriasis samples and 85 non-lesion samples), GSE34248 (14 psoriasis samples and 14 non-lesion samples), GSE41662 (24 psoriasis samples and 24 non-lesion samples), and GSE14905 (33 psoriasis samples and 28 non-lesion samples). Additionally, the single-cell dataset GSE150672 included 4 cases of leprosy, 5 cases of psoriasis, and 3 cases of normal controls (Table S1).

During the process of data integration, five datasets (GSE13355, GSE30999, GSE34248, GSE41662, GSE14905) detecting psoriasis gene expression using the GPL570 chip were integrated. Genes expressed in all samples were retained for further analysis. Batch effects were eliminated using the Combat function in the sva package^29^.

The national age-standardized incidence rates of leprosy and psoriasis were extracted from the GBD 2019 study, encompassing data from 204 countries. To visualize the data, world maps were created using the ggmap R package.

### Identification of DEGs in psoriasis and leprosy

The R package DEseq2 was utilized to identify differentially expressed genes (DEGs) in psoriasis vs. normal and leprosy vs. normal, respectively. The criteria for selection were FDR < 0.05 and |Log2FC|> 1.

### Candidate mutually exclusive genes signaling pathway enrichment analysis and PPI interaction network

The R software clusterProfiler^30^ was employed to analyze the enrichment of gene ontology (GO) terms and Kyoto Encyclopedia of Genes and Genomes (KEGG) signaling pathways for the candidate mutually exclusive genes. The STRING database (https://string-db.org/) was used to construct the PPI network, and interactions with a medium confidence score greater than 0.4 were considered statistically significant. Subsequently, Cytoscape was used to visualize the PPI network. The GeneMANIA database (https://genemania.org/) was utilized to identify critical mutually exclusive genes interaction patterns.

### Internal validation

For the E-MTAB-10318 and GSE54456 datasets, receiver operating characteristic (ROC) curves for the hub genes were generated using the pROC package^31^. Additionally, the area under the curve (AUC) was calculated for each ROC curve. Box plots were created to compare the expression of hub genes between the case and the normal control group using appropriate software packages. Pathway and gene set enrichment analysis was also conducted using Hetionet v1.0^32^.

### External validation

GSE74481 (66 leprosy and 9 normal control samples) from the GEO database was selected as the external validation set. The GSE13355, GSE30999, GSE34248, GSE41662, and GSE14905 datasets (214 psoriasis and 209 normal control samples) were integrated as part of the validation set. ROC curves associated with the hub genes were generated based on gene expression in the external validation set. Box plots were used to further illustrate the differences in gene expression between the case and the normal group.

### Transcripts-based isoform expression analysis

Raw reads were processed with Trimmomatic (v0.39) to trim low-quality reads and remove adapters. The resulting filtered reads were then aligned to the Homo sapiens (GRCh38) genome using HISAT2 (v2.2.1). Transcripts were identified and quantified utilizing StringTie (v2.2.0) against the Homo_sapiens.GRCh38.84.gtf annotation file (ftp://ftp.ensembl.org/pub/release-84/gtf/homo_sapiens/Homo_sapiens.GRCh38.84). The R package DEseq2 was utilized to identify differentially expressed transcript (DET). Raw counts were subsequently normalized using the vst function in the DESeq2 package. The difference in transcript expression between two groups was assessed using the Wilcoxon rank-sum test.

### Immune infiltration analysis

Using mRNA expression profiles normalized by DEseq2, single-sample gene set enrichment analysis (ssGSEA) in the R software GSVA package was employed to quantify the abundance of 28 immune cell types in psoriasis and leprosy samples. The cor.test function was used to calculate the correlation between hub genes expression levels and abundance of immune cells.

### Single-cell data analysis

The expression matrix GSE150672 was obtained from the GEO database. Quality control and dimensionality reduction were performed using the Seurat software package (v4.1.1). During the initial quality control step, Seurat objects were created for the normal and psoriasis groups, and genes expressing < 200 were filtered out. Genes expressed in fewer than 3 cells were also excluded. The remaining cells' gene expression profiles were normalized, and 2000 hypervariable genes were identified from each sample using the VST method. All genes were scaled, and principal component analysis was performed. Unsupervised clustering (resolution = 0.5) was used to cluster the cells, and the top 20 principal components were visualized using Uniform Manifold Approximation and Projection (UMAP). Cell type annotation was performed using the singleR package (v1.8.1), with manual annotation for optimization. Single-cell level-based gene set enrichment analyses were integrated with Hub Genes using irGSEA (v1.1.2), with the enrichment score calculation method set to UCell.

## Results

### Global epidemiology

**Figure 1:**
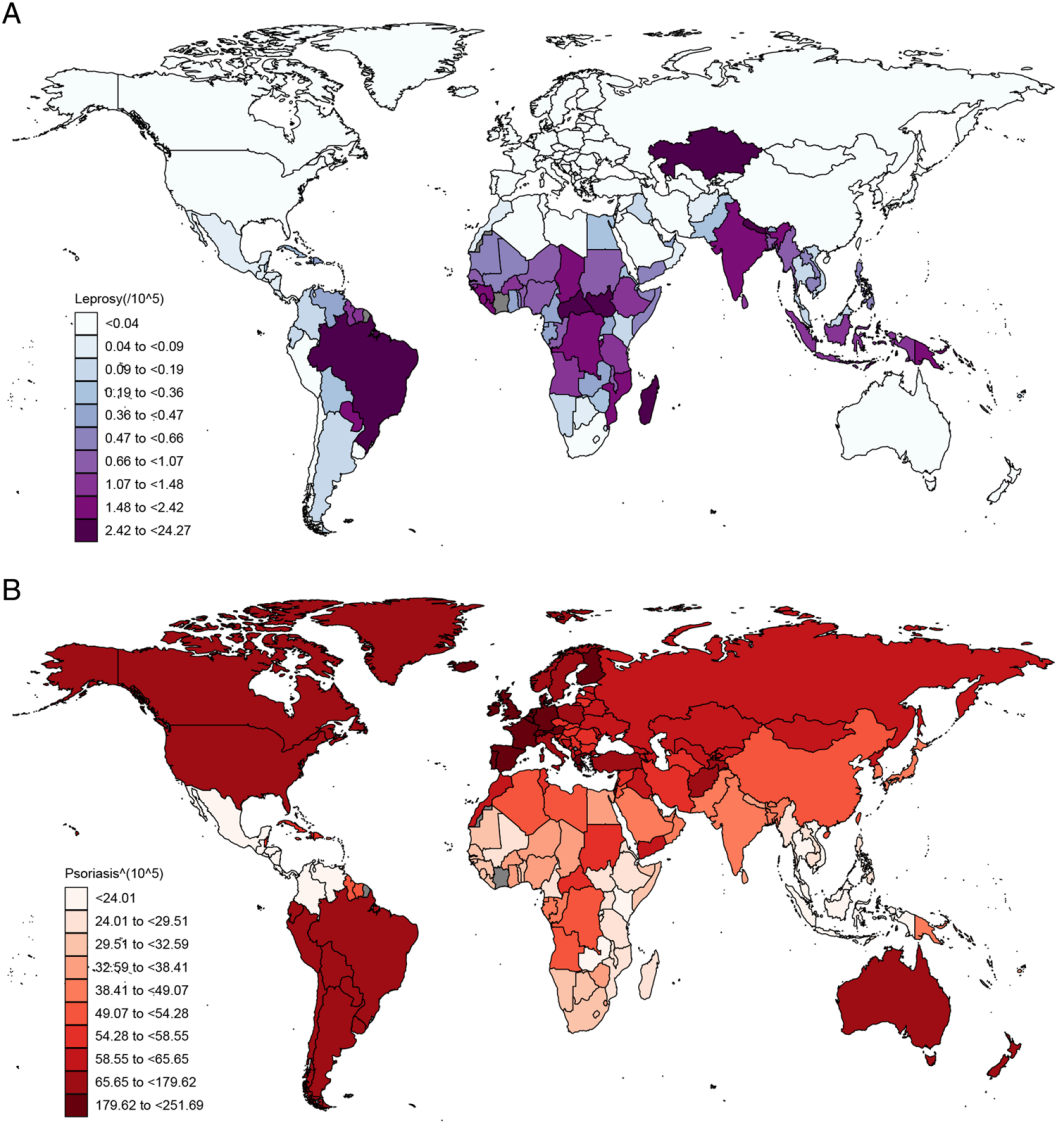
Incidence estimates of leprosy and psoriasis per 100,000 population by country in 2019. (**A**) Incidence of leprosy in 2019; (**B**) Incidence of psoriasis in 2019.

Age-standardized incidence rates per 100,000 population for leprosy varied from 0 to 24.3 cases in 2019 people (Fig. 1A). Age-standardized incidence rates per 100,000 population for psoriasis varied from 12.9 to 251.7 cases in 2019(Fig. 1B). There was an inverse association between the incidence rates of leprosy and psoriasis in the majority of countries.

### Identification of DEGs

**Figure 2:**
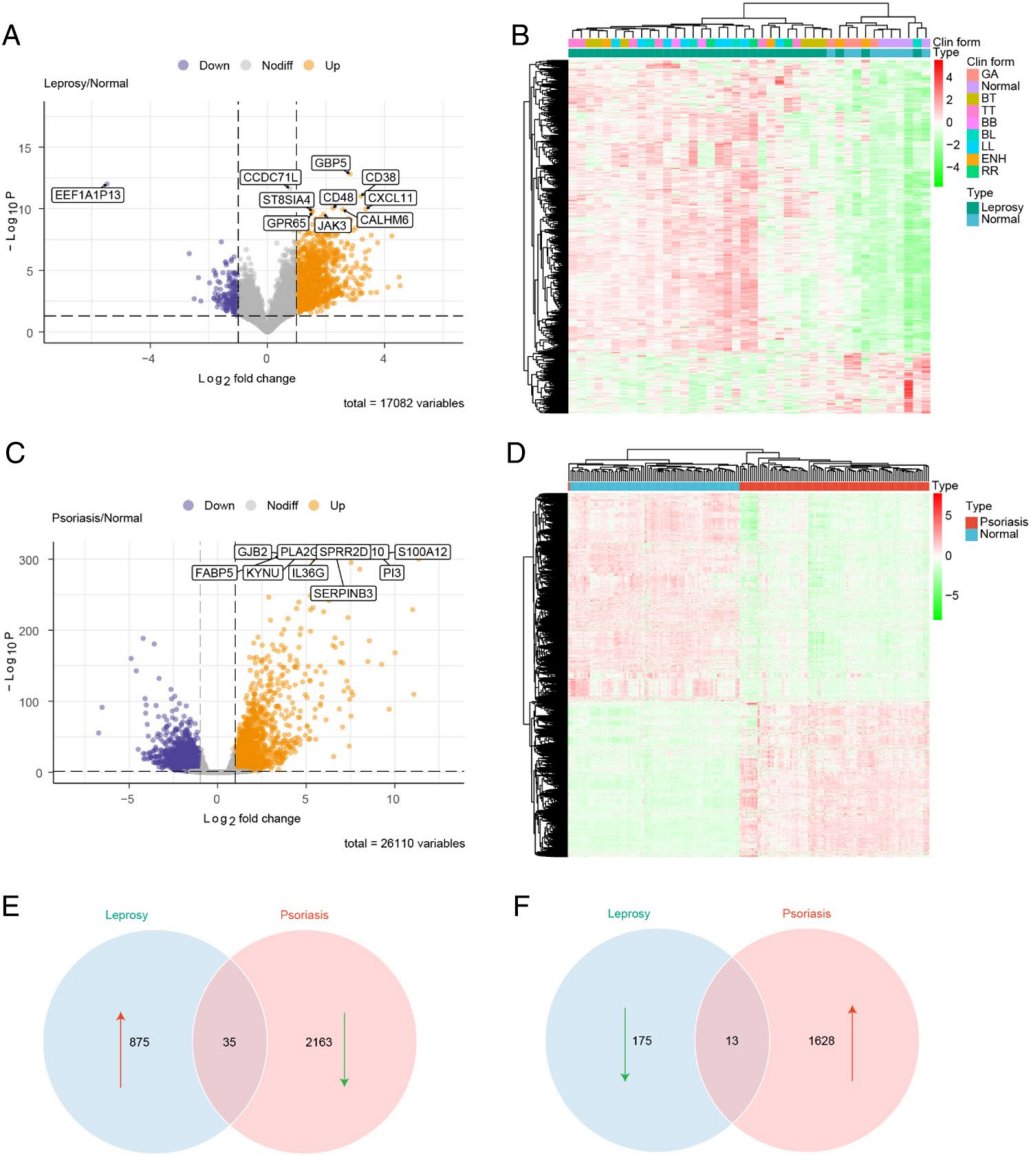
Results of differential expression analysis. (**A**) Volcano plot of differential expression in leprosy; (**B**) Heatmap of differentially expressed genes in leprosy; (**C**) Volcano plot of differential expression in psoriasis; (**D**) Heatmap of differentially expressed genes in psoriasis; (**E**) Venn diagram of up-regulated genes in leprosy and down-regulated genes in psoriasis; (**F**) Venn diagram of down-regulated genes in leprosy and up-regulated genes in psoriasis.

A total of 1098 DEGs were identified in leprosy transcriptome data, with 910 genes upregulated and 188 genes downregulated (Fig. 2A) (Table S2). Clustering analysis based on the expression levels of DEGs showed that most leprosy samples clustered together (Fig. 2B). In psoriasis transcriptome data, 3839 DEGs were identified, with 1641 genes upregulated and 2198 genes downregulated (Fig. 2C) (Table S3). Clustering analysis based on the expression levels of DEGs showed that psoriasis samples clustered together (Fig. 2D).

To identify mutually exclusive critical genes between psoriasis and leprosy, we took the intersection of genes with opposite expression direction in the two diseases. 48 candidate mutually exclusive genes were identified by taking the intersection. Among them, 35 genes were upregulated in leprosy and downregulated in psoriasis (Fig. 2E), while 13 genes were downregulated in leprosy and upregulated in psoriasis (Fig. 2F).

### Pathway enrichment analysis and PPI interaction network of candidate mutually exclusive genes

**Figure 3:**
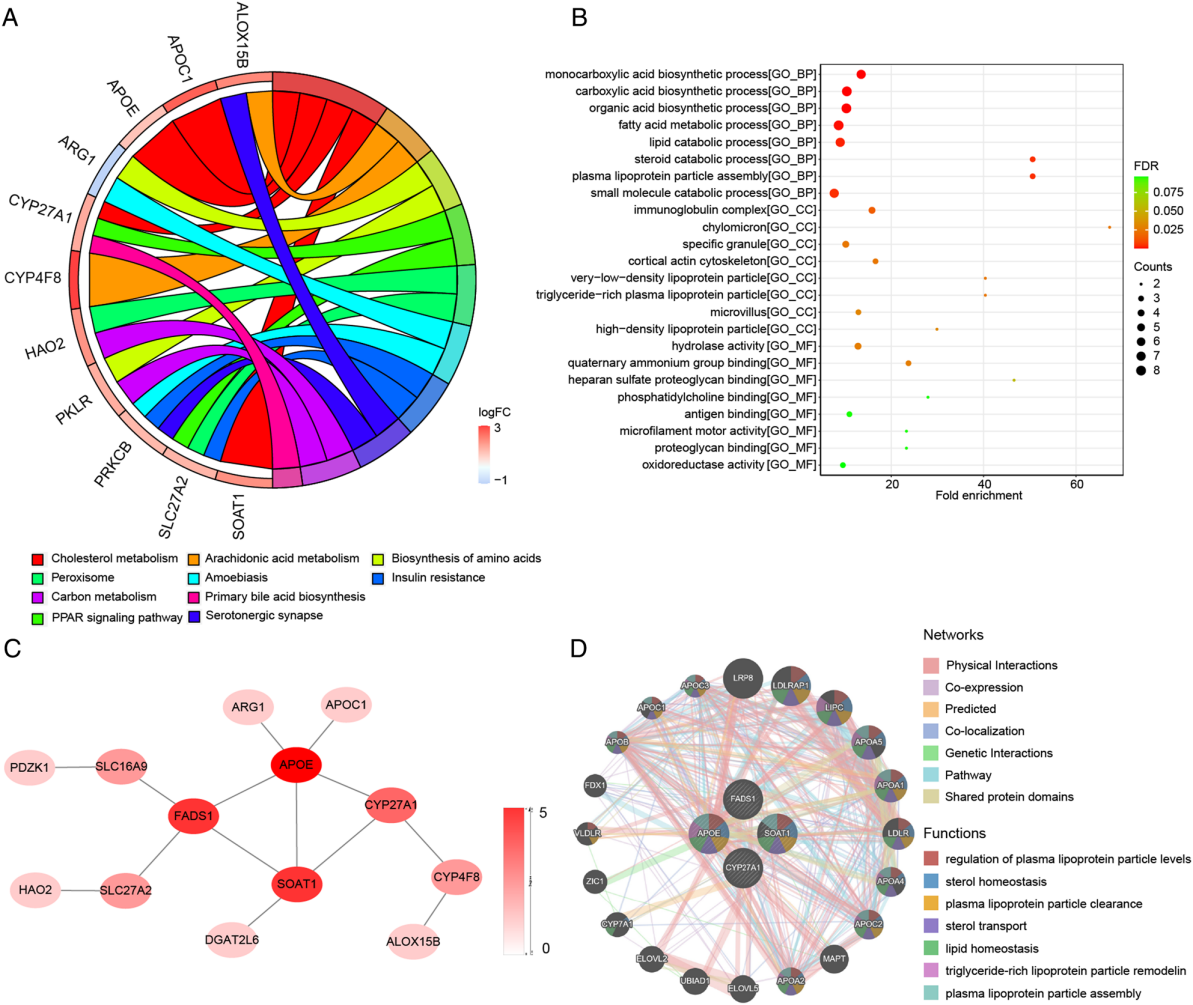
Pathway enrichment and PPI network. (**A**) Chord diagram of KEGG enrichment analysis; (**B**) Bubble diagram of GO enrichment analysis; (**C**) PPI network diagram; (**D**) hub genes interaction diagram.

We performed KEGG and GO enrichment analysis on the 48 candidate mutually exclusive genes. Enriched pathways were selected based on adj. p. value < 0.05 and count > 1, resulting in 10 enriched KEGG pathways, including cholesterol metabolism, arachidonic acid metabolism, amino acid biosynthesis, insulin resistance, synaptic vesicle, etc. (Fig. 3A) (Table S4). Additionally, 24 GO terms were enriched, including biological processes (BP), cellular components (CC), and molecular functions (MF), such as monocarboxylic acid biosynthetic process, steroid esterification, specific granule, etc. (Fig. 3B) (Table S5).

Then, 48 candidate mutually exclusive genes were used to construct a PPI network using the STRING database. The genes within this network mainly involved in vitamin K metabolic process, Lipoprotein metabolism (Table S6). APOE, CYP27A1, FADS1, and SOAT1 were identified as hub genes after applying the degree filter. (Fig. 3C). Further analysis of these genes using the GeneMANIA database revealed their involvement in processes such as regulation of plasma lipoprotein particle levels, sterol homeostasis, clearance of plasma lipoprotein particles, etc. (Fig. 3D).

### Internal validation

**Figure 4:**
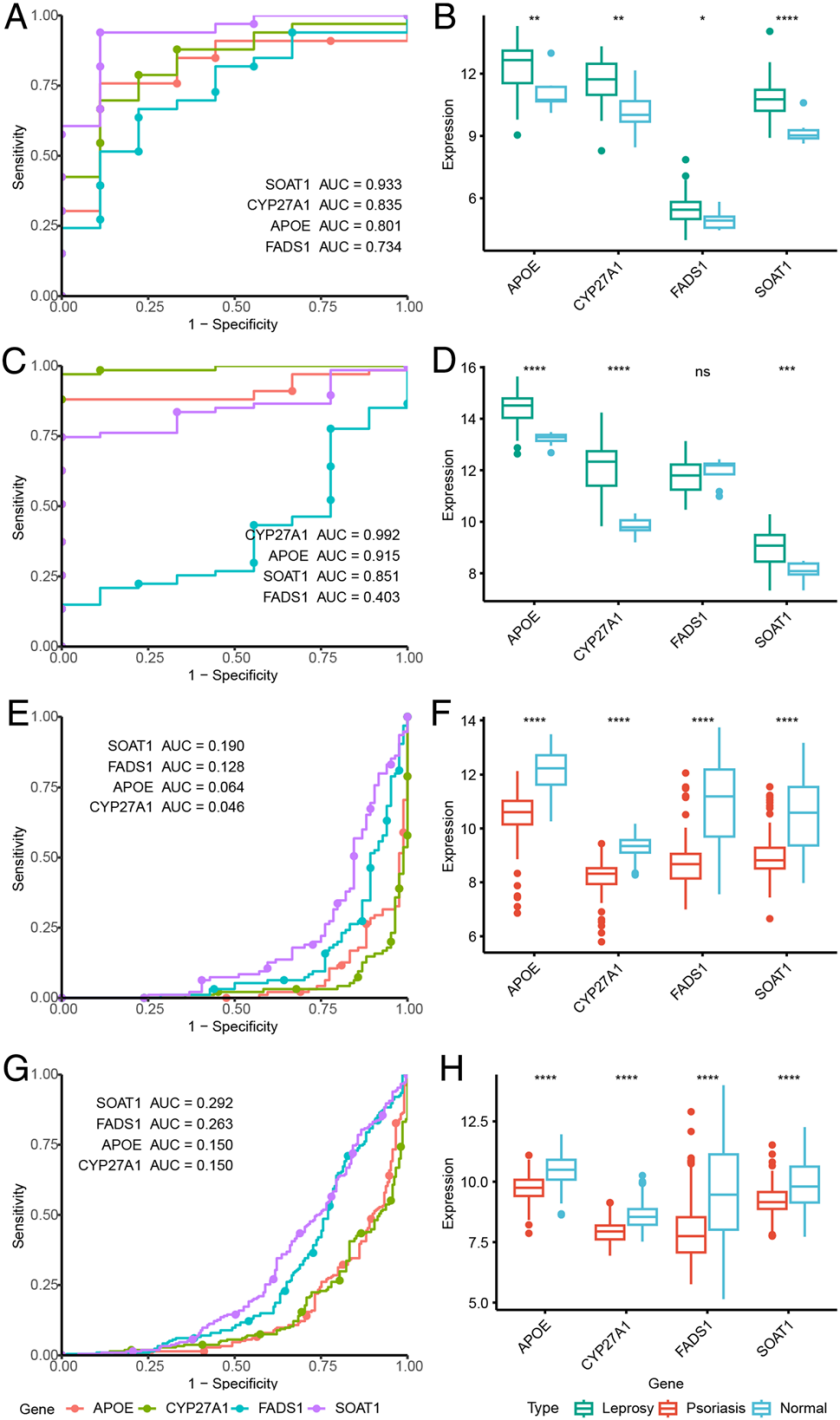
ROC curve and expression level of hub genes. (**A**) ROC curve of 4 genes in E-MTAB-10318; (**B**) Expression level of 4 genes in E-MTAB-10318; (**C**) ROC curve of 4 genes in GSE74481; (**D**) Expression level of 4 genes in GSE74481. (**E**) ROC curve of 4 genes in GSE54456; (**F**) Expression level of 4 genes in GSE54456; (**G**) ROC curve of 4 genes in psoriasis integrated dataset; (**H**) Expression levels of 4 genes in the psoriasis integrated dataset.

In the E-MTAB-10318 dataset, APOE, CYP27A1, FADS1, and SOAT1 had AUC percentages of 80.1%, 83.5%, 73.4%, and 93.3%, respectively (Fig. 4A). In the GSE54456 dataset, APOE, CYP27A1, FADS1, and SOAT1 had AUC percentages of 6.4%, 4.6%, 12.8%, and 19.0%, respectively (Fig. 4E). In the E-MTAB-10318 dataset, APOE, CYP27A1, FADS1, and SOAT1 showed higher expression levels in leprosy compared to the control group (Fig. 4B). In the GSE54456 dataset, APOE, CYP27A1, FADS1, and SOAT1 showed lower expression levels in psoriasis compared to the control group (Fig. 4F).

### External validation

In order to verify the robustness and credibility of our result, leprosy GSE74481 dataset, psoriasis integrated dataset and GSE66511 were used for external validation. Based on the expression trends and AUC values across all datasets, APOE, CYP27A1, and SOAT1 were determined to be critical mutually exclusive genes. The ROC curves showed that APOE, CYP27A1, and SOAT1 had high AUC percentages in the GSE74481 dataset (reaching 99.2%, 91.5%, and 85.1%, respectively) and low AUC percentages in the psoriasis integrated dataset (reaching 15.0%, 15.0%, and 29.2%, respectively) (Fig. 4C and G). Box plots showed APOE, CYP27A1, and SOAT1 was upregulated in leprosy tissues, whereas their expression was downregulated in psoriasis tissues (Fig. 4D and H). In GSE66511 dataset, APOE, CYP27A1, FADS1, and SOAT1 were also significantly downregulated in psoriasis (Fig. S1A). GSE66511 displayed the significant diagnostic value of those genes in psoriasis (Fig. S1B).

### Analysis of transcripts-based isoform expression

**Figure 5:**
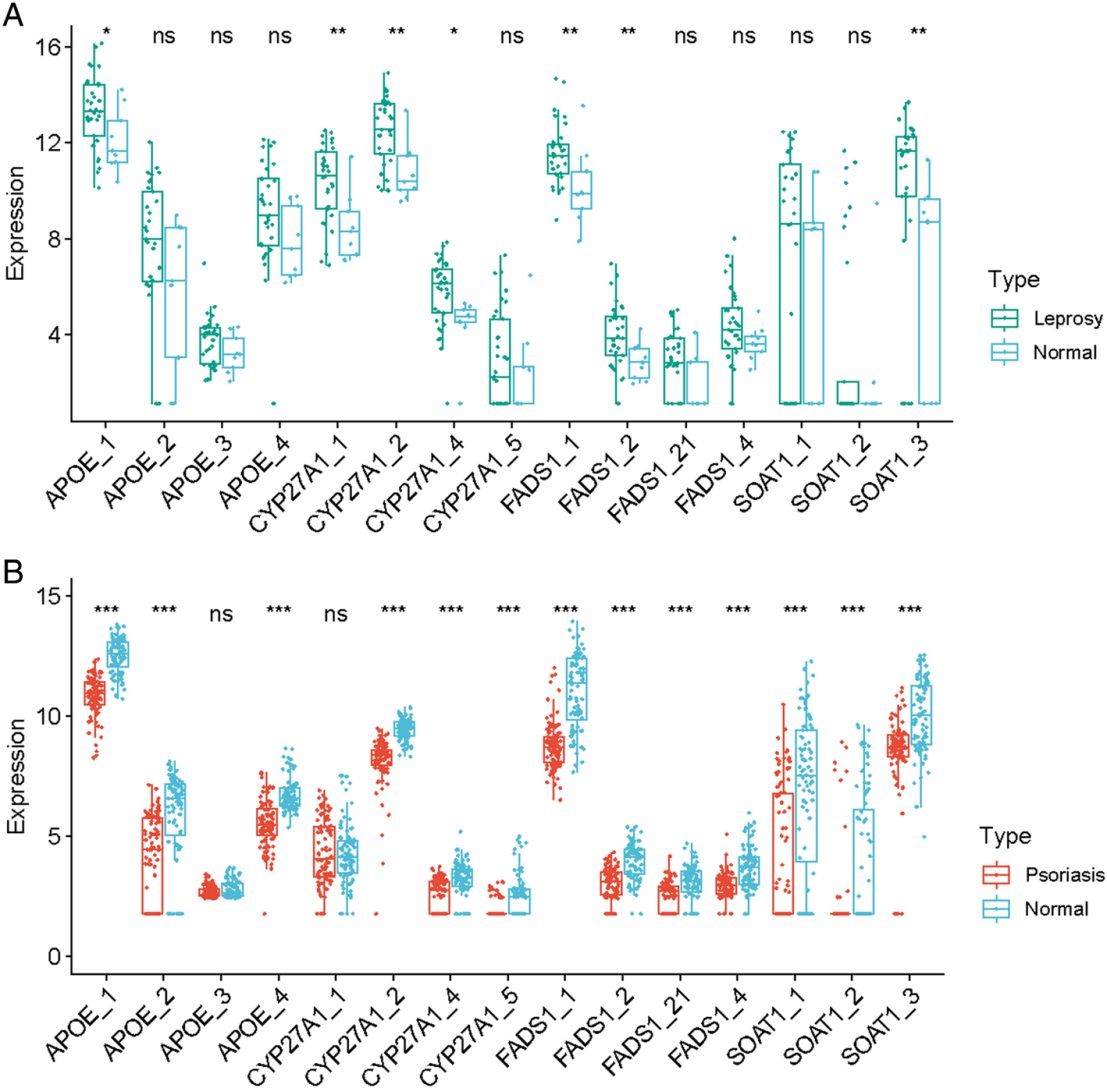
Expression level of hub genes transcripts. (**A**) Expression level of 4 hub genes transcripts in E-MTAB-10318, (**B**) Expression level of 4 hub genes transcripts in GSE54456.

A total of 2887 DETs were identified in leprosy transcriptome data, with 2556 transcripts upregulated and 331 transcripts downregulated (Table S7). In psoriasis transcriptome data, 16,160 DETs were identified, with 6892 genes upregulated and 9268 genes downregulated (Table S8). Candidate Mutually Exclusive Genes transcripts-based isoform were investigated. In the E-MTAB-10318 dataset, APOE_1(ENST00000252486), CYP27A1_1(ENST00000494263), CYP27A1_2(ENST00000258415), CYP27A1_4(ENST00000466602), FADS1_1(ENST00000350997), FADS1_2(ENST00000536991) and SOAT1_3(ENST00000367619) showed higher expression levels in leprosy compared to the control group (Fig. 5A). In the GSE54456 dataset, APOE_1, APOE_2, APOE_4, CYP27A1_2, CYP27A1_4, CYP27A1_5, FADS1_1, FADS1_2, FADS1_21, SOAT1_1, SOAT1_2 and SOAT1_3 showed lower expression levels in psoriasis compared to the control group (Fig. 5B). APOE_1, CYP27A1_2, CYP27A1_4, FADS1_1, FADS1_2 and SOAT1_3 exhibit opposite expression trends in psoriasis and leprosy group.

### Analysis of immune cell infiltration

**Figure 6:**
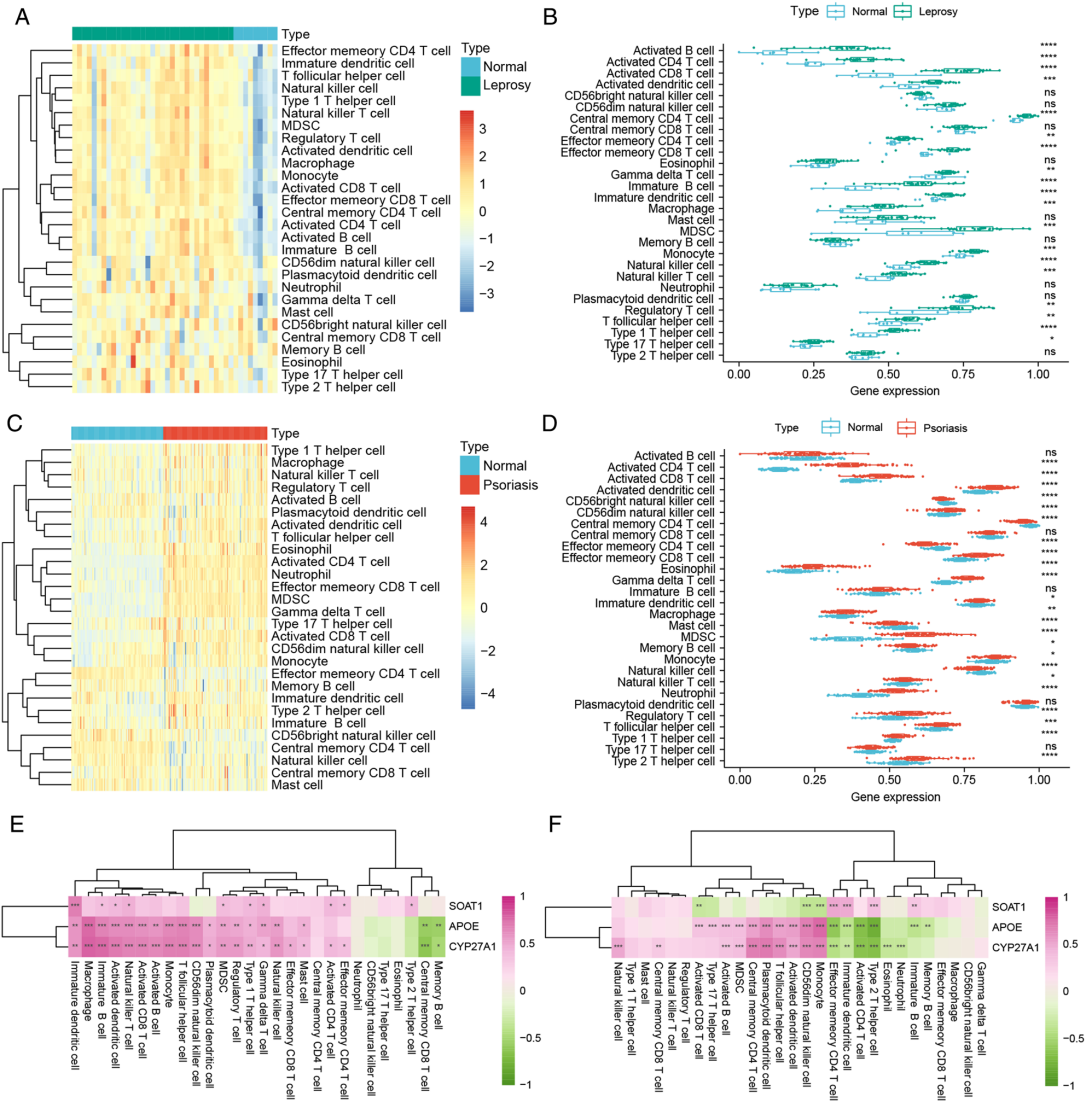
Analysis of immune cell infiltration in leprosy and psoriasis. (**A**) Heatmap of immune infiltration in leprosy; (**B**) Boxplot of differences in immune infiltration between leprosy and normal controls group; (**C**) Heatmap of immune infiltration in psoriasis; (**D**) Boxplot of differences in immune infiltration between psoriasis and normal controls group; (**E**) Correlation between critical mutually exclusive genes expression levels and immune cell abundance in leprosy; (**F**) Correlation between critical mutually exclusive genes expression levels and immune cell abundance in psoriasis.

Subsequently, we quantified immune cell infiltration in leprosy and psoriasis using ssGSEA. The results showed that the level of immune cell infiltration in leprosy was generally higher compared to normal control samples (Fig. 6A). Among the 28 types of immune cells analyzed, 19 showed higher infiltration levels in leprosy compared to normal controls (Fig. 6B). Similarly, the level of immune cell infiltration in psoriasis was generally higher than in normal control samples (Fig. 6C), with 23 out of 28 immune cell types exhibiting higher infiltration levels in psoriasis compared to normal controls sample (Fig. 6D). Notably, 16 immune cell types showed higher infiltration levels in both leprosy and psoriasis samples compared to normal controls.

Furthermore, we investigated the correlation between critical mutually exclusive genes expression levels and immune cell infiltration levels in psoriasis and leprosy. In leprosy, the expression levels of APOE and CYP27A1 were positively correlated with the levels of 18 immune cell types. Memory B cells and central memory CD8 T cells showed a negative correlation with the expression levels of APOE and CYP27A1. SOAT1 exhibited a positive correlation with 8 immune cell types (Fig. 6E). In psoriasis, APOE expression levels showed positive correlations with the levels of 10 immune cell types and negative correlations with 6 immune cell types. CYP27A1 expression levels were positively correlated with 10 immune cell types and negatively correlated with 6 immune cell types. SOAT1 expression levels exhibited a positive correlation with 4 immune cell types and a negative correlation with 3 immune cell types (Fig. 6F).

### Analysis of critical mutually exclusive genes expression at single cell level

**Figure 7:**
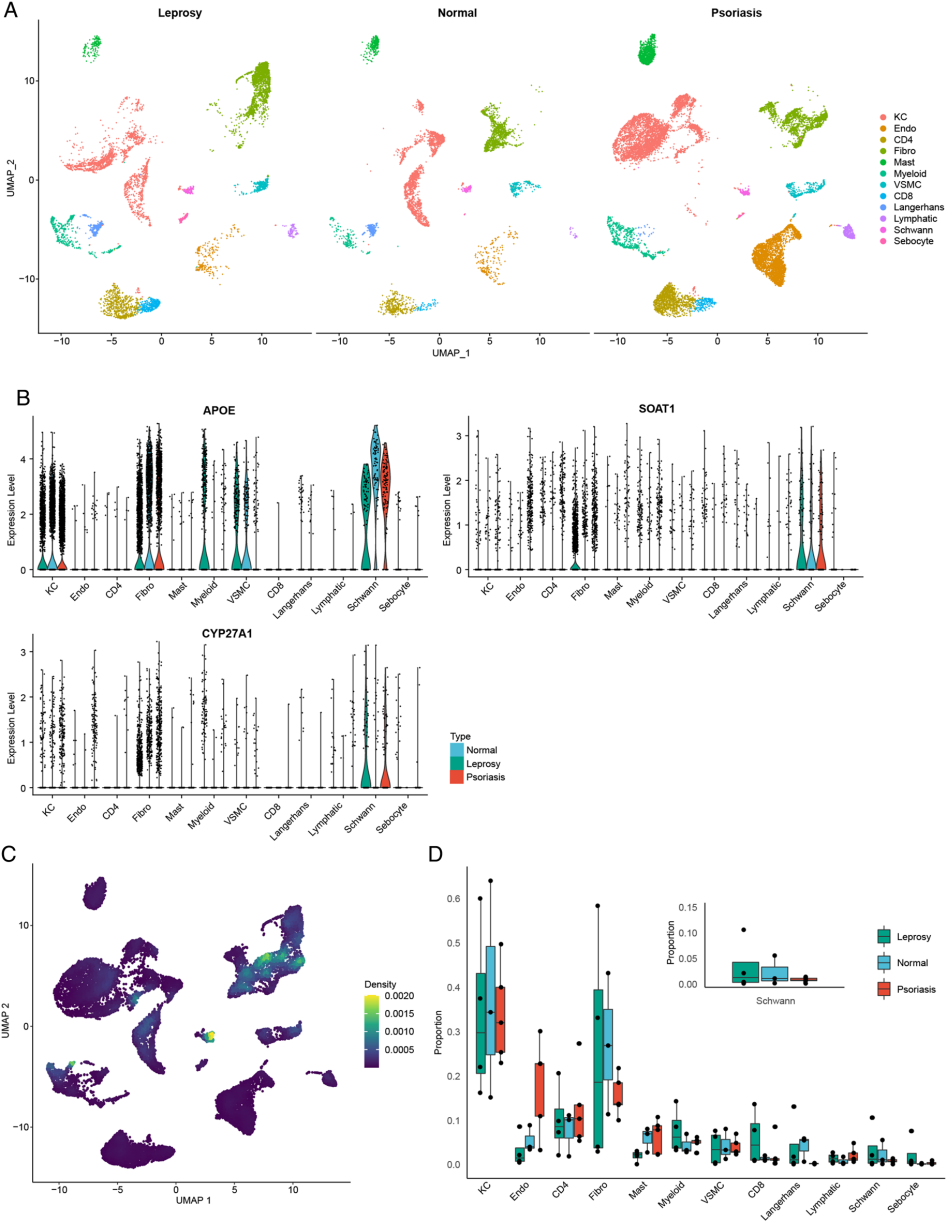
Critical mutually exclusive genes expression at single-cell level. (**A**) UMAP representation of cell distribution from psoriasis, leprosy and normal skin; Clusters are distinguished by different colors, with the identity of each cell cluster shown on the right. (**B**) Critical mutually exclusive genes expression in different types of cells. (**C**) Density scatterplot of critical mutually exclusive genes set; (**D**) The proportion of each type of cell is different among each group.

We analyzed the expression of critical mutually exclusive genes in different cell clusters in the single-cell transcriptome dataset. After cell annotation, a total of 12 cell types were identified across the samples (Fig. 7A). The marker gene of 12 cell types was list in Table S9. The top 10 marker gene of Fibro and KC were highly expressed in isolated fibroblasts and keratinocytes cell, respectively, in dataset GSE94655 (Fig. S2). The critical mutually exclusive genes were mainly expressed in Schwann cells (Fig. 7B). Schwann cells and fibroblasts had the highest cell density across all samples (Fig. 7C). The proportion of Schwann cells was higher in leprosy compared to psoriasis samples (Fig. 7D).

## Discussion

Psoriasis and leprosy rarely coexist, and there is evidence of an inverse association between these two conditions in terms of epidemiology, immunology, and genetics. However, the underlying mechanism is poorly understood. Here, the first time to our knowledge, we conducted an integrated analysis of the transcriptomes of psoriasis and leprosy to identify mutually exclusive critical pathways, genes, and cells between those two conditions. Our findings revealed that the mutually exclusive genes were predominantly enriched in pathways related to cholesterol metabolism, fatty acid metabolism, and other processes. Among DEGs, APOE, CYP27A1, and SOAT1 confirmed as critical mutually exclusive genes, exhibiting opposite expression patterns in psoriasis and leprosy. Notably, these genes were mainly expressed in Schwann cells. Our study sheds light on potential mechanisms underlying the mutual exclusion between psoriasis and leprosy, providing a foundation for future research in this field.

By analyzing the transcriptomes of psoriasis and leprosy, we identified 45 genes with opposite expression trends in the two diseases. Functional enrichment analysis revealed that they are involved in the cholesterol metabolism pathway. Previous studies have indicated higher cholesterol concentration in psoriatic lesions compared to normal controls^33^, and the accumulation of cholesterol in keratinocytes inhibits cholesterol and fatty acid biosynthesis^34^. Additionally, *M. leprae* can alter host lipid metabolism, facilitating infection and bacterial persistence^35,36^. Cholesterol concentration in leprosy lesions is also elevated compared to normal controls^37^. Previous studies have shown upregulation of genes associated with cholesterol and fatty acids in leprosy^38^. Thus, cholesterol metabolism may exhibit a mutually exclusive pattern in psoriasis and leprosy.

Four hub genes APOE, CYP27A1, FADS1 and SOAT1 were downregulated in psoriasis, while were upregulated in leprosy. When validating hub genes in an external dataset, three critical mutually exclusive genes were identified, namely APOE, CYP27A1, and SOAT1. Six isoforms from 4 hub genes exhibit opposite expression trends in psoriasis and leprosy groups. The isoform accounting for the majority of the hub gene expression belongs to these six isoforms. We observed that identical genes and their specific RNA variants are expressed differentially in both conditions, aligning with the findings of the Kõks et al.^39^.

All three critical mutually exclusive genes were involved in cholesterol metabolism pathway. Among them, APOE encodes apolipoprotein E, which plays a crucial role in lipoprotein transport and lipid metabolism^40^. APOE downregulation has been observed in psoriatic lesions, contributing to the proliferation of epidermal keratinocytes and the formation of psoriatic plaques^41,42^. On the other hand, APOE has been associated with leprosy^43^. Single-cell transcriptome sequencing has showed APOE upregulated in leprosy lesions^44^. Plasma lipoproteins elevation promote the survival of *M. leprae*^36^.

CYP27A1 encodes cytochrome P450 oxidase, which is involved in the synthesis of cholesterol, steroids, and other lipids^45^. Yu et al.^46^ found CYP27A1 was downregulated in psoriasis. CYP27A1 hydroxylates cholesterol^47^ and elevated cholesterol levels in psoriatic lesional skin play a critical role in IL-17A signaling and suppress cholesterol and fatty acid biosynthesis genes^34^. While, CYP27A1 in *M. leprae* host cells may oxidize cholesterone produced by *M.* *leprae,* regulating host cell functions and promoting the invasion and persistence of *M. leprae*^48^.

SOAT1 encodes Sterol O-Acyltransferase 1, which is essential for maintaining cellular cholesterol homeostasis^49^. SOAT1 was downregulation in psoriasis skin lesions^50^, The function SOAT1 in psoriasis and leprosy has not been extensively studied. SOAT1 expression level was positively correlated with macrophages, neutrophils, Th17 cells, and activated dendritic cells, while negatively correlated with plasmacytoid dendritic cells or natural killer cells in glioma^51^. Inhibition of SOAT1 expression can have anti-inflammatory effects by altering free cholesterol levels or oxysterol levels^52^.

We further analyzed the differential immune cells between leprosy and normal control samples, as well as psoriasis and normal control samples. The infiltration levels of 16 immune cell types were found to be higher in both leprosy and psoriasis samples compared to normal controls. Transcriptome analysis of psoriasis demonstrated a significant increase in γδT cells, resting NK cells, M0 macrophages, M1 macrophages, activated dendritic cells, and neutrophils in the skin^53,54^. While, immune cell infiltration in leprosy has not been extensively reported. Correlation analysis between APOE, CYP27A1, SOAT1, and immune cells revealed positive associations between the expression levels of APOE and CYP27A1 and the abundance of six immune cell types in both psoriasis and leprosy patients. Recent studies have shown that peptides derived from human APOE exhibit immunomodulatory and antibacterial effects^55^. CYP27A1 is highly expressed in bone marrow immune cells and macrophages and promotes breast cancer by impairing T cell expansion^56^.

At the single-cell level, the expression levels of APOE, CYP27A1, and SOAT1 were found to be highest in Schwann cells and fibroblasts. Schwann cells not only support repair and promote axonal regeneration in the peripheral nervous system but also contribute to the dissemination of *Bacillus leprae* in leprosy patients^57^. The increased cholesterol in leprosy patients leads to lipid accumulation in Schwann cells in the form of lipid droplets, regulating inflammation and immune responses^35^. Leprosy involves peripheral nerves at some point during the course of the disease^58^, while patients with psoriasis may experience remission following nerve injury^28^.

Furthermore, mutually exclusive genes were found to be enriched in lipoprotein metabolism and the vitamin K metabolism pathway. Notably, vitamin K has been recognized for its role in sphingolipid formation^59^. Sphingolipid regulate a diverse range of processes such as cell proliferation, differentiation, inflammation, endocytosis, and neural transmission. For instance, in psoriasis, elevated levels of S1P can inhibit the proliferation of keratinocytes involved in the formation of the stratum corneum and promote their differentiation, S1PR1 modulators can ameliorate psoriasis^60,61^. *Mycobacterium leprae*, the causative agent of leprosy, is capable of synthesizing sphingolipids to enhance its entry into host cells^62^. The bacterium interacts with specific sphingolipids on the cell membrane, potentially facilitating its invasion of macrophages and Schwann cells^63,64^ and, consequently, leading to infection. In the context of leprosy, the bacterium can infect Schwann cells, resulting in damage to peripheral nerves^65^. Schwann cells are acknowledged for their sphingolipid production^66^, and their infection may disrupt the delicate balance of sphingolipids in nerve cells, potentially contributing to nerve damage. In addition, *Mycobacterium leprae* has been demonstrated to exert influence on host immune responses by altering the host's sphingolipid metabolism^67^, thereby impacting the immune system's efficacy in combating the infection.

In summary, our study utilized transcriptome data to elucidate the mutually exclusive mechanisms underlying leprosy and psoriasis. Notably, APOE, CYP27A1, and SOAT1 identified as candidate mutually exclusive genes between the two diseases, with their involvement in cholesterol metabolism pathways. These findings provide a theoretical basis for further research in this field and contribute to our understanding of the mutual exclusion between leprosy and psoriasis at a molecular level. However, it is important to note that our study has limitations, such as the limited information available in public databases and the reliance on bioinformatics analysis. Further experimental verification is necessary to confirm our findings, despite the support from previous studies.

## Conclusion

Current bioinformatics studies have provided valuable insights into the transcriptomic profiles of leprosy and psoriasis, revealing potential mutually exclusive genes between these two diseases. Among these genes, APOE, CYP27A1, and SOAT1 have been identified as critical mutually exclusive genes and are associated with cholesterol metabolism pathways. These findings suggest the molecular mechanism that contributes to the mutual exclusion of leprosy and psoriasis, providing a foundation for further research in this field.

### Supplementary Information

Supplementary Figure S1.

Supplementary Figure S2.

Supplementary Table S1.

Supplementary Table S2.

Supplementary Table S3.

Supplementary Table S4.

Supplementary Table S5.

Supplementary Table S6.

Supplementary Table S7.

Supplementary Table S8.

Supplementary Table S9.

## Data Availability

Statistical analyses were performed using the R 4.2.0 software. All data generated or analyzed during this study are included in this published article. The gene expression profiles and clinical data can be found at the GDC portal and GEO (https://www.ncbi.nlm.nih.gov/geo/) and the ArrayExpress (https://www.ebi.ac.uk/biostudies/arrayexpress/) database. Software and resources used for the analyses are described in each method section. All R scripts and data generated or analyzed during this study are available from the corresponding author on reasonable request.
