# Supplementary figures and images for: Identification of gene signatures and molecular mechanisms underlying the mutual exclusion between psoriasis and leprosy
Source: Sci Rep. 2024 Jan 25;14:2199. doi: 10.1038/s41598-024-52783-0 (PMC10810956; doi:10.1038/s41598-024-52783-0)

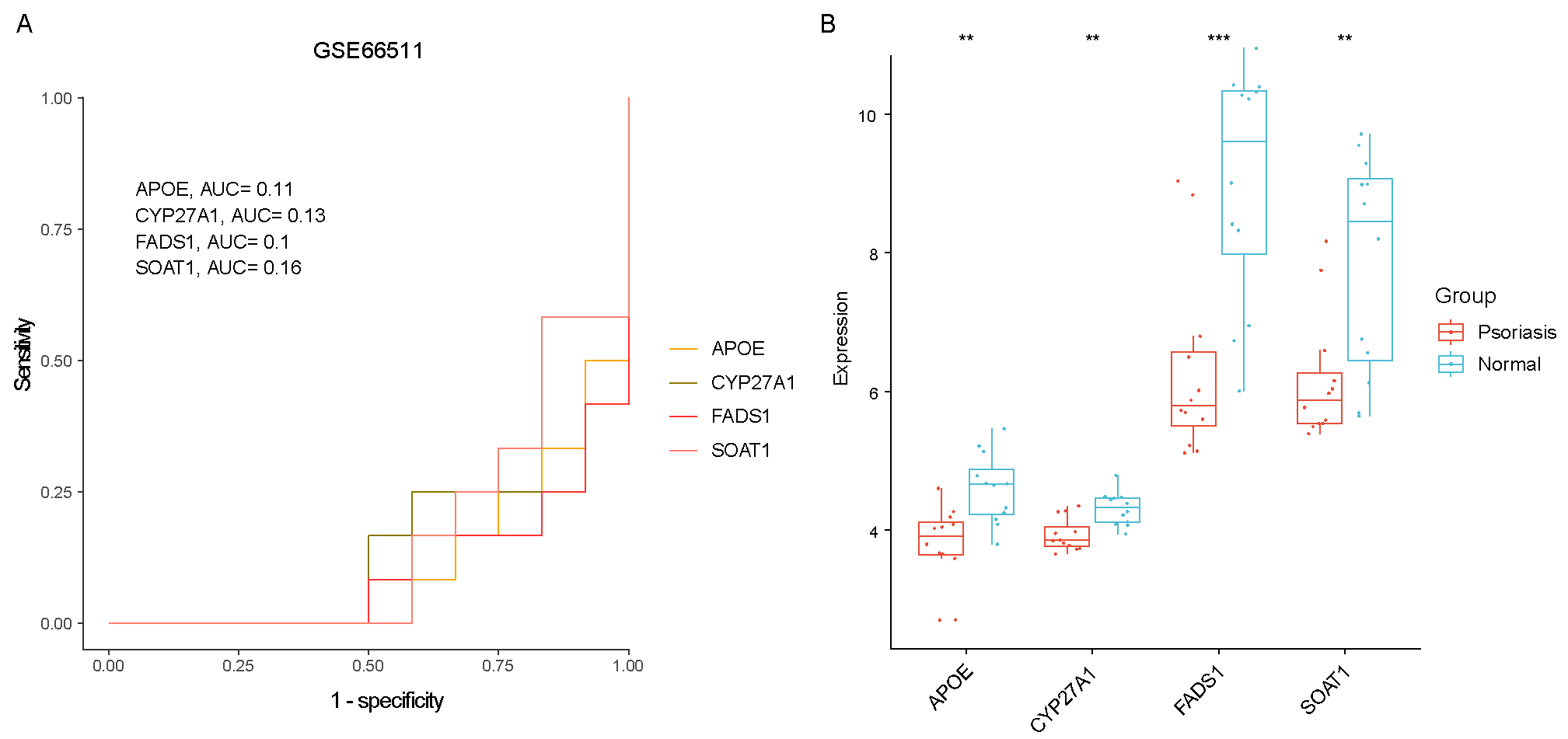

Supplement: Supplementary file 1 — Supplementary Figure S1. [file 41598_2024_52783_MOESM1_ESM.tif]

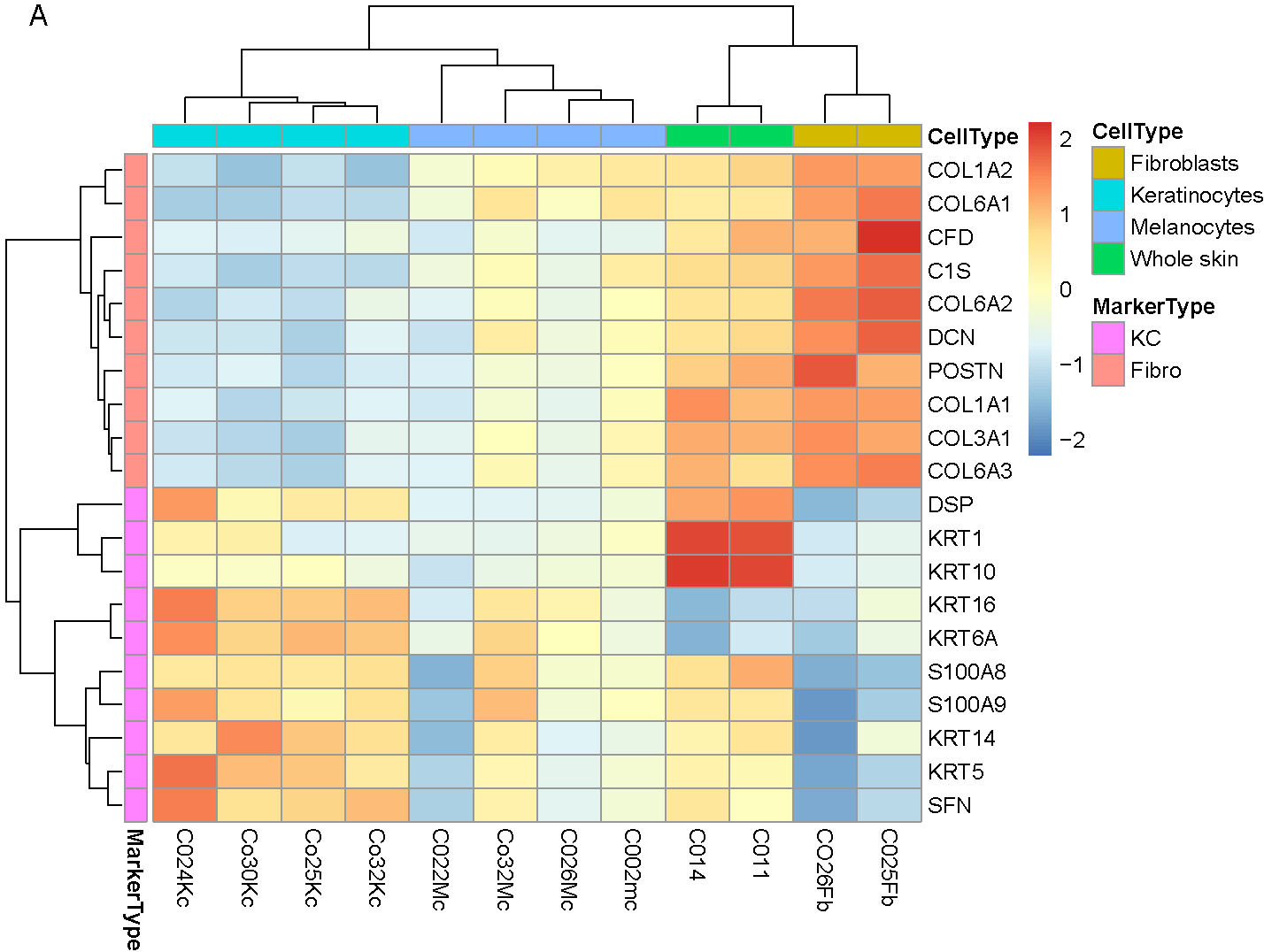

Supplement: Supplementary file 2 — Supplementary Figure S2. [file 41598_2024_52783_MOESM2_ESM.tif]
